# A flexible protocol for a systematic review of remote patient monitoring

**DOI:** 10.1017/S1463423620000262

**Published:** 2020-10-15

**Authors:** Ashley Elizabeth Muller, Rigmor C. Berg

**Affiliations:** 1 Department of Reviews and Health Technology Assessments, Norwegian Institute of Public Health (NIPH), Oslo, Norway; 2 Department of Community Medicine, The Arctic University of Norway, University of Tromsø (UiT), Tromsø, Norway

**Keywords:** chronic disease, remote patient monitoring, systematic review

## Abstract

**Background:**

Norway is interested in implementing remote patient monitoring (RPM) within primary health services. This systematic review will first identify the types of RPM that are of interest to Norwegian health authorities, then synthesize the effects of RPM on clinical health and health service utilization outcomes among adults with chronic diseases.

**Methods:**

We will perform systematic literature searches in multiple databases, using RPM-related searches, such as telemedicine, telemonitoring, and eHealth. Based on what research exists, the review will be selected from a cascading menu of review types. Methodological quality will be assessed through appropriate checklists, while the quality of the evidence will be assessed through Grading of Recommendations Assessment, Development, and Evaluation.

**Discussion:**

This flexible protocol specifies the production of different possible types of reviews of RPM. It is anticipated that the results of the review will inform the development of effective RPM programs to the most appropriate chronic disease groups.

## Introduction

### What is the problem?

Norway, as many other industrialized countries, has an aging population with increasing burdens of chronic diseases. While this increased longevity is the positive result of advances in medical technology that prevent mortality from acute diseases, chronic conditions continue to accumulate with age. Sixty-year-old Norwegians can expect to live another 22 years, and 10 of those years will be burdened by morbidity from chronic diseases (Knudsen *et al*., 2017).

The preferred approach to long-term management of chronic diseases is within a chronic care model, in which patients receive well-coordinated, flexible, and proactive care, driven by regular assessments (Wagner *et al*., 2001; Helse- og omsorgsdepartement, 2013; WHO, 2016). Regular assessments after the onset of chronic diseases are crucial to monitor treatment progress, prevent deterioration, and prevent the development of additional diseases, injuries, and complications. With more frequent patient assessments, there is more accurate and individualized treatment decisions; with real-time data, patients and providers are more likely to be aware of the need for health services *before* deterioration (Wagner *et al*., 2001). However, frequent face-to-face meetings with health care providers are burdensome for both the patient and the provider and are often neither prioritized nor feasible, especially for lower-risk patients.

Unfortunately, there is little evidence that chronic diseases are being managed within a chronic care model: from 2014 to 2017, the amount of preventable hospitalizations among chronically ill Norwegians increased by 5% from 2014 to 2017 (Helsedirektoratet, 2019). The Directorate of Health (DoH) has suggested that appropriate follow-up of these patients by their general practitioners could help prevent overuse of hospital and other specialist health services (Helsedirektoratet, 2019). Other countries such as the United Kingdom and New Zealand have also begun focusing on managing patients with complex or multiple chronic conditions in primary care (Adan *et al*., 2020; McGeoch *et al*., 2019).

### What is the solution?

Ideally, patients could transmit health data without seeing providers, and this data could be sent and evaluated often enough to initiate interventions or treatment adjustments before the patient’s health status becomes acute. Remote patient monitoring (RPM; also referred to as telemonitoring, remote care, telehealth, home monitoring, and telerehabilitation) uses these strategies. The collection and transmission of data outside the traditional points of care contact allow patients to remain at home while receiving follow-up and to receive follow-up only if their condition warrants it. This should increase equality in health care access for patients living in rural areas and patients with physical, economic, or other mobility barriers.

Given the increasing prevalence of chronic conditions in the Norwegian population along with an increasing amount of unnecessary specialist health services utilization, it is not feasible to expect that specialist health care providers will be able to provide the type of frequent, preventative, and non-acute monitoring that many patients could benefit from. The government has therefore prioritized piloting RPM as well as other types of chronic care and care coordination programs within the primary health services, administrated by the municipalities (Helse- og omsorgsdepartement, 2013; Helse- og omsorgsdepartement, 2015; Helsedirektoratet, 2017). The intention is to curb preventable and unnecessary consumption of specialist health services, such as hospital and emergency room admissions, to situate primary care providers in the center of chronic disease management and to allow people to remain at home and within their social networks. The Norwegian DoH has developed a specific definition of RPM that describes exactly the type of processes that they consider are most relevant for Norway. This definition is described further in the *Methods*, but briefly, data are transmitted from a non-institutionalized patient to a provider remotely, and the provider evaluates the data manually and contacts the patient, or the data are automatically evaluated (i.e., by the device), but providers are contacted for follow-up if values are concerning.

### Why do we need this systematic review?

It is unclear whether the large amount of research on monitoring, broadly defined, is applicable to Norway, given the specific type of RPM that the DoH is interested in further developing. For example, one recent overview of 19 systematic reviews (SRs) of the effect on heart failure outcomes concluded that ‘remote monitoring’ strategies reduce mortality (Bashi *et al*., 2017). However, the overview included mobile phone applications that did not involve providers. This is a type of remote monitoring that does not meet the DoH definition of RPM. Another overview of four SRs of ‘telehealth remote patient monitoring’ among patients with type 2 diabetes found a statistically significant, but small, reduction in blood glucose levels (Lee *et al*., 2018). This overview also included automated programs and therefore does not meet the DoH definition of RPM. Lastly, a 2014 overview identified 13 reviews of ‘telemonitoring’ of chronic disease patients served in primary care, but with telemonitoring not further defined (Purcell *et al*., 2014). In our planned SR, we will carefully assess the types of RPM identified against the DoH definition of RPM in order to synthesize evidence that is most relevant and applicable to Norway. We will also examine the extent to which the reviews and their meta-analyses provide coherent, mutually supportive evidence, based on the idea that such evidence is stronger than incoherent/inconsistent evidence (Mickenautsch, 2012).

### Objectives

This is a protocol for aSR. We have three objectives:

1. Identify overviews of SRs, and SRs, that report clinical and health service utilization outcomes following RPM of chronically ill patients in the primary health care sector.
2. Summarize the evidence on the effect of RPM on the health status of chronically ill patients and their consumption of health and care services.
3. Explore the coherence of SRs and meta-analyses on the effects of RPM.

## Methods

### Review types

The DoH definition of RPM is specific enough that existing overviews and SRs may not meet inclusion criteria. This protocol therefore specifies five potential review types, based on the search results, and described according to the PRISMA-P checklist, available in Appendix 1 (Shamseer *et al*., 2015). In this section, we provide a brief description of the procedure, while further details follow in the sections below.

**Figure 1.**
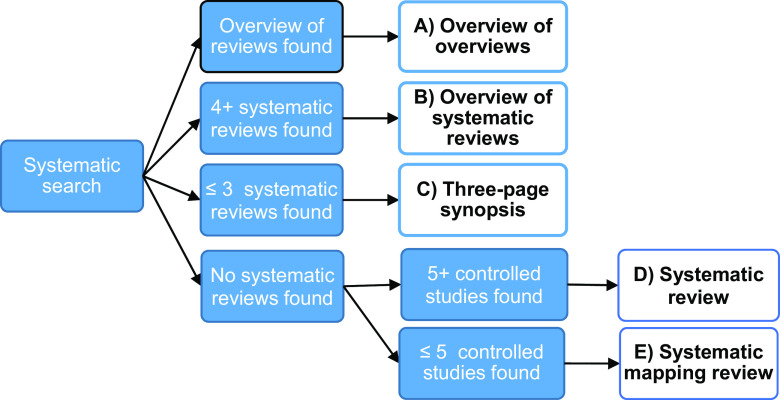
Potential review types based on the search results.

As shown in Figure 1, the type of review we conduct will be determined by the existing research. The order of priority is from the top down, that is, if the search results in one or more overview of SRs that we assess as having high methodological quality (see Quality evaluation below), we will conduct an overview of overviews (review type A). If no overview of reviews are found but we instead find four or more SRs, we will write an overview of SRs (Review B). If we find fewer than four SRs, we will write a three-page synopsis of each of these (Review C). If we do not identify overviews of SRs or SRs that meet the inclusion criteria, that is, if we lack research to conduct alternative A, B, or C, we will search for primary studies. If we find at least five high-quality randomized controlled trials (RCTs), non-randomized controlled trials (NRCTs), or controlled before-and-after studies (CBAs), we will conduct an SR of these (Review D). If we do not find at least five of the above controlled studies, we will perform a systematic mapping (scoping) review of effect studies (review type E).

### Eligibility

#### Inclusion criteria

- Types of participants: Persons who are 18 years or older, and who are neither in the very early or very advanced stage of one or more of the following: cardiovascular disease, diabetes, chronic lung diseases, cancer, mental disorders, chronic musculoskeletal disorders, osteoporosis, or impaired vision/hearing; and persons who are neither in the very early nor very acute phase of these conditions.
- Types of interventions: Bidirectional RPM in which patient health data are transmitted remotely and are then evaluated by a health care provider in the primary health services (e.g., general practitioners, municipal health services, home care services). Alternatively, data evaluation can be automated, as long as the provider will be looped in if data are of concern. RPM must be provided within the primary health services. Devices can be telephones, mobile telephones, videos, portable devices, or implantable devices.
- Types of comparators: Standard care that does not involve RPM; or other type of RPM.
- Primary outcomes: mental health (symptoms or diagnoses); diagnosis-specific physical health; physical functioning level; quality of life; consumption of health services (hospital admissions, emergency care, number of bed-days, outpatient consultations, nursing home stays, home care, and general practitioner consultations); or health services costs.
- Secondary outcomes: employment; education; social health (isolation, loneliness); patient experiences; or health literacy. Other secondary outcomes may be considered if they are similar to the aforementioned secondary outcomes.
- Years: Overviews of overviews and SRs: published in 2015 and later, to ensure the capture of the most updated technologies. If no SRs are found, we will include primary studies published in 2010 and later.

We will assess ‘very early’ and ‘very advanced’ stages according to the authors’ descriptions of their populations, for example, a population recently diagnosed would be assessed as in an early stage, while inpatients receiving intensive care may be assessed as in an advanced stage. If the population is mixed, for example, both children and adults or both chronic diseases and non-chronic diseases, studies will be included as long as ≥50% meet the inclusion criteria, or if the results are presented separately so that the outcomes related to our populations of interest can be extracted. If the intervention is mixed, for example, RPM that is conducted both in primary and specialist health service settings, studies will be included as long as ≥50% meet the inclusion criteria, or if interventions are reported separately.

No languages will be excluded a priori, but studies in languages that are mastered by neither the project staff nor our colleagues will be excluded. These will be listed in an appendix.

#### Exclusion criteria

- Types of studies: Qualitative studies; non-empirical studies.
- Types of participants: Persons with reduced cognitive function who may not be able to report their own health status.
- Types of interventions: Fully automated RPM technologies that do not require input from health care providers; purely internet-based programs; mobile applications on phones or tablets; treatment provided at a distance; RPM in which the first provider involved is a specialist or working within the specialist health services.
- Outcomes: Medication adherence; treatment adherence.
- Settings: Reviews and studies that explicitly exclude Norway or the part of the world in which Norway is located, for example, studies of low- and middle-income countries.

### Methodological quality criteria

We will only include reviews that are of high methodological quality. To our knowledge, there is not yet a quality or reporting checklist for overviews of overviews. If we conduct Review A, we will modify our research center’s [Norwegian Institute of Public Health (NIPH)] existing checklist for SRs, as suggested by recent research regarding this lack of quality standards (Ballard and Montgomery, 2017, Hunt *et al*., 2018). To be assessed as high quality in the NIPH checklist, reviews must demonstrate a clear research question, adequate search design, appropriate analysis of results and assess the quality of the original studies. If we conduct Review B or C, we will use the NIPH checklist for SRs (based on the Center for Evidence-based Medicine checklist). If we conduct Review D, we will use the Cochrane Risk of Bias tool for RCTs (Higgins *et al*., 2011), and Cochrane’s Effective Practice and Organisation of Care checklist for NRCTs and CBA trials. Two researchers will independently assess methodological quality, and disagreements will be resolved through discussion. A third researcher will be engaged if disagreements cannot be resolved.

### Search strategy and study selection

We recognize that a wide range of terms are in use to describe what we have operationalized as RPM, but we expect that many studies using the term RPM will not meet our inclusion criteria. We have therefore developed a comprehensive search strategy, available from the authors upon request, that also includes terms such as telehealth, home monitoring, remote care, eHealth, and mHealth. An information specialist will perform the electronic database search, in the following databases: Cochrane Library, Epistemonikos, MEDLINE (OVID), EMBASE, and Web of Science. Two researchers will independently review titles and abstracts for potentially relevant studies. Conflicts will be resolved through discussion. The full texts of potentially relevant studies will be obtained and read independently by two researchers, with a third researcher available to resolve conflicts.

### Data extraction, synthesis, and presentation

One researcher will perform data extraction and another will check her extraction, using Covidence software. In Reviews A, B, and C, we will use the results provided by the review authors. We will group data from the reviews by chronic disease type and summarize the evidence presented. The following data will be extracted from the included reviews: title, author, research question, time period of the search, number of included studies (and participants), study designs included, methodological quality, population and context, details of RPM, comparison(s), outcomes, and results of the reviews.

If no overviews or SRs are identified, we will redo our search to include primary studies. At least five included controlled studies (RCTs, NRCTs, or CBAs) will result in a SR (Review D). If the majority are assessed as having high methodological quality, as described in *Methodological quality criteria*, we will only extract data from these. The following data will be extracted: title, author, study design, context, number of and characteristics of participants, RPM details, comparison, outcomes, and results. Results of each outcome will be reported separately and grouped by chronic disease. We will analyze the dichotomous outcome measures by calculating the relative risk and the 95% confidence interval (CI). We will analyze continuous outcomes using the mean difference with 95% CI or standardized mean difference, if the outcome measures have different units or scales of measurements. Results from different study designs will be analyzed separately. We will perform meta-analyses if primary studies have the same outcomes and are sufficiently similar in terms of population, intervention, comparison, and effect measurement. Random effects models will be used, given our expectation that RPM will have different effects in different contexts and with different populations (Borenstein *et al*., 2009). We will use the Mantel–Haenszel method for dichotomous outcomes and the inverse-variance method for continuous outcomes. We will evaluate statistical heterogeneity with Chi-square test and I-square values. Subgroup analyses will be conducted if possible on: participants with single chronic diseases versus multiple, follow-up by health personnel versus non-health personnel, and different types of RPM.

Preliminary searches suggest we will find studies that allow us to conduct Review A, B, C, or D. We therefore consider Review E unlikely. Nevertheless, if we do not identify five or more controlled studies, we will conduct a systematic mapping review that provides an overview of the empirical research available regarding our research question. The results of a mapping review will be based on the same literature search as for Review D and will be summarized in text and tables. The methodological framework suggested by Arksey and O’Malley (2005) and further developed by Levac *et al*. (2010) will guide the mapping review. This framework includes the following steps: after identification of the research question, study selection, and data extraction, data will be sorted and summarized in close consultation with the commissioner. Data summaries are simpler than in SRs; in exchange, the summary of results will be highly tailored to the DoH’s interests and needs for information.

### Assessment of the quality of evidence in included studies

The Grading of Recommendations Assessment, Development, and Evaluation (GRADE) method is a tool to assess confidence in up to seven of the most frequently reported primary outcomes. If we conduct Review A, B, or C, we will use the review authors’ own GRADE assessments, if available. If it is not available, we will use information from the reviews to perform a GRADE assessment. If we conduct Review D, we will perform a GRADE assessment. We will use study design as a starting point and then consider the following five criteria for each outcome measure: methodological study quality, degree of consistency, directness, dissemination bias, and precision of data. Upgrading is possible for outcomes from observational studies if there is a large effect estimate, a dose-response gradient, or if all plausible effect modifiers, if present, would reduce the effect. More descriptions of how we use GRADE to assess confidence in results can be found in Guyatt *et al*. (2011). Certainty of the evidence is not assessed in mapping reviews (Review E).

## Discussion

This flexible protocol is for what may be the first SR of RPM strategies among chronic disease patients that uses a definition of RPM developed by a national commissioning organization, so as to be most relevant to national health policy priorities. One of five possible SR types will be conducted, according to the results of the literature search. Results of the review will describe the clinical efficacy of bidirectional RPM administered in the primary health services, for up to eight different groups of chronic disease patients. Separating results by chronic disease group will help policy-makers prioritize RPM for the groups in Norway that are most likely to benefit. We expect that results will also be informative for the Norwegian DoH as it moves forward with recommendations to municipal health services. Any gaps we identify in the evidence can also inform future implementation research.
